# First record of the non-native *Osbornellusauronitens* (Provancher, 1889) (Hemiptera, Cicadellidae, Deltocephalinae) in Italy

**DOI:** 10.3897/BDJ.11.e106166

**Published:** 2023-07-04

**Authors:** Enrico Ruzzier, Federico Lessio, Francesca Cinquatti, Francesco Poggi, Alberto Alma, Andrea Galli, Luciano Bani, Francesco Sanna

**Affiliations:** 1 NBFC, National Biodiversity Future Center, Palermo, Italy NBFC, National Biodiversity Future Center Palermo Italy; 2 Department of Science, Roma Tre University, viale G. Marconi 446, Rome, Italy Department of Science, Roma Tre University, viale G. Marconi 446 Rome Italy; 3 Regione Piemonte - Direzione Agricoltura - Settore Fitosanitario e Servizi Tecnico-Scientifici, Turin, Italy Regione Piemonte - Direzione Agricoltura - Settore Fitosanitario e Servizi Tecnico-Scientifici Turin Italy; 4 Dipartimento di Scienze Agrarie Forestali e Alimentari (DISAFA), Grugliasco (TO), Italy Dipartimento di Scienze Agrarie Forestali e Alimentari (DISAFA) Grugliasco (TO) Italy; 5 Independent researcher, Missaglia, Italy Independent researcher Missaglia Italy; 6 Department of Earth and Environmental Sciences, University of Milano-Bicocca, Milan, Italy Department of Earth and Environmental Sciences, University of Milano-Bicocca Milan Italy; 7 Department of Agronomy, Food, Natural Resources, Animals and the Environment (DAFNAE), Legnaro (Padova), Italy Department of Agronomy, Food, Natural Resources, Animals and the Environment (DAFNAE) Legnaro (Padova) Italy

**Keywords:** alien species, biodiversity, distribution, Europe, leafhoppers

## Abstract

**Background:**

Globalisation and international trade, in particular, are the major drivers of introduction and the spread of non-native species. To date, more than 30 species of non-native Hemiptera Auchenorrhyncha have been accidentally introduced into Europe. Some species are invasive with important repercussions primarily for agricultural activities, while almost no information exists on their impacts within natural ecosystems. Therefore, early detection of non-native species and their subsequent monitoring are extremely important actions to undertake.

**New information:**

The North American *Osbornellusauronitens* (Provancher, 1889), firstly recorded for the Palearctic and Europe in Switzerland in 2016, is recorded in Italy for the first time on the basis of 77 specimens collected between August 2015 and October 2022.

## Introduction

Over the past fifty years, more than 30 species of leafhoppers, planthoppers and allies (Hemiptera, Auchenorrhyncha) have been introduced into Europe (D’Urso et al. 2019, Sanna and Poggi 2022), sometimes having a substantial negative impact for agriculture (e.g. Chuche (2014), Bocca et al. (2020), Duso et al. (2020), Prazaru et al. (2021)). The damage caused is often due not only to the trophic activity of nymphs and adults, but also to the transmission of plant pathogens (Weintraub and Beanland 2006).

The genus *Osbornellus* Ball, 1932 (Cicadellidae, Deltocephalinae, Scaphoideini) includes more than 100 described species, subdivided into four subgenera (Linnavuori 1959, Dlabola 1967): *Osbornellus* Ball, 1932, *Sorbonellus* Linnavuori, 1959, *Nereius* Linnavuori, 1959 and *Mavromoustaca* Dlabola, 1967. Species within *Osbornellus* (*s. str.*) are equally distributed all over the Nearctic and Neotropical realms (Beamer 1937, DeLong and Martinson 1976, Dominguez and Godoy 2010) and includes species possessing the apical gonopore and having the pygofer lacking thorn-like process; *Sorbonellus*, known from the Neotropics (Linnavuori 1959, Dominguez and Godoy 2010), includes species with a subapical gonopore and the pygofer without a thorn-like process; *Nereius*, known from the Caribbean Region (Freytag 2008, Dominguez and Godoy 2010), includes species with a claw-like stylus, the outer subapical cell of the forewing small and the pygofer without a thorn-like process; *Mavromoustaca*, that host species with pygofer having a thorn-like process, is represented only by a few species restricted to the Old World (Lindberg 1948, Dlabola 1974, Dlabola 1984, Dlabola 1987a, Dlabola 1987b, Li et al. 2011). Very little is known about the biology of *Osbornellus* (*s. str.*), but it is generally believed that the species of this genus are polyphagous, capable of developing on both herbaceous and woody plants (Wilde 1962, Gibson 1973).

During both recent trapping activities aimed at collecting Coleoptera in Lombardy (NE Italy) and monitoring activities finalising the survey of the invasive *Scaphoideustitanus* Ball, 1932 and other Cicadellidae of phytosanitary interest in Piedmont (NW Italy), a species of *Osbornellus*, extraneous to the Italian fauna, was recorded. The species is here identified as the Nearctic *Osbornellusauronitens* (Provancher, 1889), representing the third record of this species in the Palaearctic (Europe) (see Trivellone et al. (2017), Trivellone et al. (2022)) and the first one in Italy. In addition, the revision of previously unidentified material revealed the species presence in Italy since at least 2015.

## Materials and methods

The single specimen, upon which the DNA barcoding is based, was collected using a bottle trap, baited with 80% ethanol intended to attract beetles (Ruzzier et al. 2021). The trap was located inside a mixed forest (broadleaves and conifers). Other specimens suspected to belong to the genus *Osbornellus* Ball, 1932 were collected in 2022 on yellow sticky traps (YST) placed in 12 vineyards and two apple orchards for monitoring other leafhoppers within the framework of other research projects and/or survey activities conducted by the Regional Plant Protection Service of Piedmont. Traps (Glutor®, Biogard, Grassobbio, BG, Italy) were 40 x 25 cm in size and were hung on the wires of grapevine/apple tree rows. In each orchard/vineyard, three traps were placed, arranged in a diagonal pattern. Traps were set at the beginning of July and replaced every two weeks until the middle of October. When changed, traps were wrapped into a plastic film, labelled and freezer preserved. The specimens from Lombardy, other than that used for DNA barcoding, were collected at light. Morphological identification was based on male genitalia; the genital capsule was carefully dissected and placed in a 10% potassium hydroxide solution to remove membranaceous soft tissues. Thereafter, species attribution was done by using published taxonomic keys and related literature (e.g. DeLong (1941), Martinson (1977), Trivellone et al. (2017)). Specimens collected with sticky traps were removed with tweezers and a drop of xylene and stored in Eppendorf® for 16 hours. Samples were subsequently washed twice in absolute ethanol to remove any residual xylene and dry-mounted.

Specimen repository (acronym): FPPC; Francesco Poggi private collection (Missaglia, Italy); FSPC: Francesco Sanna private collection (Bovolone, Italy); DISAFAc: Dipartimento di Scienze Agrarie, Forestali e Alimentari (DISAFA) collection, Università degli Studi di Torino (Grugliasco, Italy).

Genital structure images were taken with a DeltaPix digital camera mounted on a Laboval-4 microscope. Adult specimen photographs were taken with a DeltaPix digital camera mounted on an Optech-GZ 808 stereomicroscope. The digital images were then imported into DeltaPix InSight 6.4.7.0 (64-bit) for stacking, Gimp 2.10.8 for clipping and Magix Photo & Graphic Designer 19 for plate composition. The measurements were taken with DeltaPix InSight 6.4.7.0 software. Body length was measured from the apex of the vertex to the tip of the forewings.

Molecular identification was based on the DNA barcode. DNA extraction, purification and cytochrome c oxidase subunit I (COX1) amplification followed the methodology described in Ruzzier et al. (2020). PCR products were purified using Exonuclease and Antarctic Phosphatase (GE Healthcare) and sequenced at the BMR Genomics Service (Padova, Italy). The sequence was edited using MEGA XI (Tamura et al. 2021) and subsequently, translated with Transeq (EMBOSS) to exclude the presence of stop codons in the coding region. The obtained barcode was deposited in GenBank under the accession number OP218770. An analysis of the obtained sequence was run through the integrated bioinformatics platform Barcode of Life Data (BOLD) System database to assess the identity of the species. To produce the most complete COX1 sequences set to be used in the phylogenetic analysis, the barcode obtained was integrated with the North American complete sequences of *O.auronitens* available in GenBank [accession numbers: KR043272, KR038961, KR042200, KR043875, KR041827, KR044796, KR031230, KR032627, KR037705, KR034871, KR032003, KR042584, KR043624, KR042310] and Bold System [accession number CNCHG897-12] and that referring to the specimens found in Switzerland [accession numbers: KY006567]. *Cicadellaviridis* (Linnaeus, 1758) (Cicadellidae, Cicadellinae; sequences numb. KC135928, LC619113, FR775764) was included in the analysis as the outgroup, being the type species of Cicadellinae, a close relative of Deltocephalinae. Evolutionary analysis was conducted using the *Phylogeny.fr: robust phylogenetic analysis for the non-specialist* server (Dereeper et al. 2008). Tree topology was evaluated using the Approximate likeihood ratio test (Anisimova and Gascuel 2006).

## Taxon treatments

### Osbornellus (Osbornellus) auronitens

(Provancher, 1889)

E9895FA9-262C-5181-B9DB-2E63B91063D5

OP218770

#### Materials

- **Type status:** Other material. **Occurrence:** recordedBy: Francesco Poggi; individualCount: 2; sex: 1 male, 1 female; lifeStage: adult; occurrenceID: 2DE2EAAB-DECA-546D-9FB5-2A6DEDA3CA9C; **Taxon:** scientificName: *Osbornellusauronitens* (Provancher, 1889); order: Hemiptera; family: Cicadellidae; genus: Osbornellus ; specificEpithet: *auronitens*; scientificNameAuthorship: (Provancher, 1889); **Location:** country: Italy; countryCode: IT; stateProvince: Lombardy; county: Lecco; municipality: Maresso; decimalLatitude: 45.689722; decimalLongitude: 9.355833; geodeticDatum: WGS84; **Identification:** identifiedBy: Francesco Poggi; **Event:** eventDate: 2015-08-02; **Record Level:** collectionID: FPPC
- **Type status:** Other material. **Occurrence:** recordedBy: Andrea Galli; individualCount: 1; sex: female; lifeStage: adult; occurrenceID: 991FA595-EB46-5555-9576-C3897FE165BD; **Taxon:** scientificName: *Osbornellusauronitens* (Provancher, 1889); order: Hemiptera; family: Cicadellidae; genus: Osbornellus ; specificEpithet: *auronitens*; scientificNameAuthorship: (Provancher, 1889); **Location:** country: Italy; countryCode: IT; stateProvince: Lombardy; county: Varese; municipality: Casorate Sempione; decimalLatitude: 45.689722; decimalLongitude: 9.355833; geodeticDatum: WGS84; **Identification:** identifiedBy: Francesco Sanna; **Event:** eventDate: 2017-07-14; **Record Level:** collectionID: FSPC
- **Type status:** Other material. **Occurrence:** recordedBy: Federico Lessio; individualCount: 4; sex: males; lifeStage: adult; occurrenceID: 6A653A09-979F-5DAC-84CC-4D234D2646AB; **Taxon:** scientificName: Osbornellusauronitens (Provancher, 1889); order: Hemiptera; family: Cicadellidae; genus: Osbornellus; specificEpithet: auronitens; scientificNameAuthorship: (Provancher, 1889); **Location:** country: Italy; countryCode: IT; stateProvince: Piedmont; county: Torino; municipality: Borgomasino/Maglione; decimalLatitude: 45.357086; decimalLongitude: 8.000192; geodeticDatum: WGS84; **Identification:** identifiedBy: Francesca Cinquatti; **Event:** eventDate: 2022-08-31; **Record Level:** collectionID: DISAFAc
- **Type status:** Other material. **Occurrence:** recordedBy: Federico Lessio; individualCount: 1; sex: female; lifeStage: adult; occurrenceID: D30F5BDF-83AB-5179-8D6C-449F80DBFC38; **Taxon:** scientificName: Osbornellusauronitens (Provancher, 1889); order: Hemiptera; family: Cicadellidae; genus: Osbornellus; specificEpithet: auronitens; scientificNameAuthorship: (Provancher, 1889); **Location:** country: Italy; countryCode: IT; stateProvince: Piedmont; county: Torino; municipality: San Martino Canavese; decimalLatitude: 45.389848; decimalLongitude: 7.820653; geodeticDatum: WGS84; **Identification:** identifiedBy: Francesca Cinquatti; **Event:** eventDate: 2022-08-31; **Record Level:** collectionID: DISAFAc
- **Type status:** Other material. **Occurrence:** recordedBy: Federico Lessio; individualCount: 1; sex: male; lifeStage: adult; occurrenceID: 358617F3-1814-5668-B2D4-68A9F56FDAB4; **Taxon:** scientificName: Osbornellusauronitens (Provancher, 1889); order: Hemiptera; family: Cicadellidae; genus: Osbornellus; specificEpithet: auronitens; scientificNameAuthorship: (Provancher, 1889); **Location:** country: Italy; countryCode: IT; stateProvince: Piedmont; county: Torino; municipality: Borgomasino/Maglione; decimalLatitude: 45.357086; decimalLongitude: 8.000192; geodeticDatum: WGS84; **Identification:** identifiedBy: Francesca Cinquatti; **Event:** eventDate: 2022-08-31; **Record Level:** collectionID: DISAFAc
- **Type status:** Other material. **Occurrence:** recordedBy: Federico Lessio; individualCount: 1; sex: male; lifeStage: adult; occurrenceID: 66364B1A-0FE6-58CD-BB64-56F2530C1145; **Taxon:** scientificName: Osbornellusauronitens (Provancher, 1889); order: Hemiptera; family: Cicadellidae; genus: Osbornellus; specificEpithet: auronitens; scientificNameAuthorship: (Provancher, 1889); **Location:** country: Italy; countryCode: IT; stateProvince: Piedmont; county: Torino; municipality: Borgomasino/Maglione; decimalLatitude: 45.357086; decimalLongitude: 8.000192; geodeticDatum: WGS84; **Identification:** identifiedBy: Francesca Cinquatti; **Event:** eventDate: 2022-08-31; **Record Level:** collectionID: DISAFAc
- **Type status:** Other material. **Occurrence:** recordedBy: Federico Lessio; individualCount: 3; sex: 2 males, 1 female; lifeStage: adult; occurrenceID: 78BF5182-AD6E-551E-AF3C-7E06BBFAF767; **Taxon:** scientificName: Osbornellusauronitens (Provancher, 1889); order: Hemiptera; family: Cicadellidae; genus: Osbornellus; specificEpithet: auronitens; scientificNameAuthorship: (Provancher, 1889); **Location:** country: Italy; countryCode: IT; stateProvince: Piedmont; county: Torino; municipality: Maglione; decimalLatitude: 45.366302; decimalLongitude: 8.005953; geodeticDatum: WGS84; **Identification:** identifiedBy: Francesca Cinquatti; **Event:** eventDate: 2022-08-31; **Record Level:** collectionID: DISAFAc
- **Type status:** Other material. **Occurrence:** recordedBy: Federico Lessio; individualCount: 3; sex: 1 male, 2 females; lifeStage: adult; occurrenceID: 34E9B423-2328-5799-87DD-8141576E7AA4; **Taxon:** scientificName: Osbornellusauronitens (Provancher, 1889); order: Hemiptera; family: Cicadellidae; genus: Osbornellus; specificEpithet: auronitens; scientificNameAuthorship: (Provancher, 1889); **Location:** country: Italy; countryCode: IT; stateProvince: Piedmont; county: Torino; municipality: Maglione; decimalLatitude: 45.366302; decimalLongitude: 8.005953; geodeticDatum: WGS84; **Identification:** identifiedBy: Francesca Cinquatti; **Event:** eventDate: 2022-09-01; **Record Level:** collectionID: DISAFAc
- **Type status:** Other material. **Occurrence:** recordedBy: Federico Lessio; individualCount: 2; sex: 1 male, 1 female; lifeStage: adult; occurrenceID: 9CA1104A-88A1-522A-85E1-2C6872EE2210; **Taxon:** scientificName: Osbornellusauronitens (Provancher, 1889); order: Hemiptera; family: Cicadellidae; genus: Osbornellus; specificEpithet: auronitens; scientificNameAuthorship: (Provancher, 1889); **Location:** country: Italy; countryCode: IT; stateProvince: Piedmont; county: Biella; municipality: Cavagliŕ; decimalLatitude: 45.418575; decimalLongitude: 8.124102; geodeticDatum: WGS84; **Identification:** identifiedBy: Francesca Cinquatti; **Event:** eventDate: 2022-09-01; **Record Level:** collectionID: DISAFAc
- **Type status:** Other material. **Occurrence:** recordedBy: Federico Lessio; individualCount: 8; sex: 5 males, 3 females; lifeStage: adult; occurrenceID: 363658F6-0A33-5F3B-B046-F0AE8EF8893A; **Taxon:** scientificName: Osbornellusauronitens (Provancher, 1889); order: Hemiptera; family: Cicadellidae; genus: Osbornellus; specificEpithet: auronitens; scientificNameAuthorship: (Provancher, 1889); **Location:** country: Italy; countryCode: IT; stateProvince: Piedmont; county: Torino; municipality: Albiano d'Ivrea; decimalLatitude: 45.433117; decimalLongitude: 7.965388; geodeticDatum: WGS84; **Identification:** identifiedBy: Francesca Cinquatti; **Event:** eventDate: 2022-09-01; **Record Level:** collectionID: DISAFAc
- **Type status:** Other material. **Occurrence:** recordedBy: Federico Lessio; individualCount: 3; sex: 2 males, 1 female; lifeStage: adult; occurrenceID: E39BE5DF-139D-52F4-AC5B-8152DDFE1A1A; **Taxon:** scientificName: Osbornellusauronitens (Provancher, 1889); order: Hemiptera; family: Cicadellidae; genus: Osbornellus; specificEpithet: auronitens; scientificNameAuthorship: (Provancher, 1889); **Location:** country: Italy; countryCode: IT; stateProvince: Piedmont; county: Torino; municipality: Palazzo Canavese; decimalLatitude: 45.461246; decimalLongitude: 7.990519; geodeticDatum: WGS84; **Identification:** identifiedBy: Francesca Cinquatti; **Event:** eventDate: 2022-09-01; **Record Level:** collectionID: DISAFAc
- **Type status:** Other material. **Occurrence:** recordedBy: Federico Lessio; individualCount: 6; sex: 3 males, 3 females; lifeStage: adult; occurrenceID: F5572402-B864-5555-A04F-2FE2857CCC9B; **Taxon:** scientificName: Osbornellusauronitens (Provancher, 1889); order: Hemiptera; family: Cicadellidae; genus: Osbornellus; specificEpithet: auronitens; scientificNameAuthorship: (Provancher, 1889); **Location:** country: Italy; countryCode: IT; stateProvince: Piedmont; county: Torino; municipality: Borgomasino; decimalLatitude: 45.365531; decimalLongitude: 7.989988; geodeticDatum: WGS84; **Identification:** identifiedBy: Francesca Cinquatti; **Event:** eventDate: 2022-09-01; **Record Level:** collectionID: DISAFAc
- **Type status:** Other material. **Occurrence:** recordedBy: Demis Del Forte; individualCount: 1; sex: male; lifeStage: adult; occurrenceID: EBFA3CE3-E775-546E-8D78-4BFE6495A47E; **Taxon:** scientificName: Osbornellusauronitens (Provancher, 1889); order: Hemiptera; family: Cicadellidae; genus: Osbornellus; specificEpithet: auronitens; scientificNameAuthorship: (Provancher, 1889); **Location:** country: Italy; countryCode: IT; stateProvince: Piedmont; county: Torino; municipality: Caluso; decimalLatitude: 45.307469; decimalLongitude: 7.910311; geodeticDatum: WGS84; **Identification:** identifiedBy: Francesca Cinquatti; **Event:** eventDate: 2022-07-16; **Record Level:** collectionID: DISAFAc
- **Type status:** Other material. **Occurrence:** recordedBy: Demis Del Forte; individualCount: 1; sex: male; lifeStage: adult; occurrenceID: F2F6702D-F1E1-5716-A42B-261A1B68CC24; **Taxon:** scientificName: Osbornellusauronitens (Provancher, 1889); order: Hemiptera; family: Cicadellidae; genus: Osbornellus; specificEpithet: auronitens; scientificNameAuthorship: (Provancher, 1889); **Location:** country: Italy; countryCode: IT; stateProvince: Piedmont; county: Torino; municipality: Caluso; decimalLatitude: 45.307469; decimalLongitude: 7.910311; geodeticDatum: WGS84; **Identification:** identifiedBy: Francesca Cinquatti; **Event:** eventDate: 2022-07-16; **Record Level:** collectionID: DISAFAc
- **Type status:** Other material. **Occurrence:** recordedBy: Federico Lessio; individualCount: 1; sex: female; lifeStage: adult; occurrenceID: 9C86001A-184C-534C-91D8-52E11012EC1C; **Taxon:** scientificName: Osbornellusauronitens (Provancher, 1889); order: Hemiptera; family: Cicadellidae; genus: Osbornellus; specificEpithet: auronitens; scientificNameAuthorship: (Provancher, 1889); **Location:** country: Italy; countryCode: IT; stateProvince: Piedmont; county: Torino; municipality: Montaldo Dora; decimalLatitude: 45.498004; decimalLongitude: 7.873812; geodeticDatum: WGS84; **Identification:** identifiedBy: Francesca Cinquatti; **Event:** eventDate: 2022-09-06; **Record Level:** collectionID: DISAFAc
- **Type status:** Other material. **Occurrence:** recordedBy: Andrea Arpellino; individualCount: 2; sex: 1 male, 1 female; lifeStage: adult; occurrenceID: 7F2CA568-8D67-515E-96D4-A0FC80692D01; **Taxon:** scientificName: Osbornellusauronitens (Provancher, 1889); order: Hemiptera; family: Cicadellidae; genus: Osbornellus; specificEpithet: auronitens; scientificNameAuthorship: (Provancher, 1889); **Location:** country: Italy; countryCode: IT; stateProvince: Piedmont; county: Torino; municipality: Viverone; decimalLatitude: 45.428387; decimalLongitude: 8.066156; geodeticDatum: WGS84; **Identification:** identifiedBy: Francesca Cinquatti; **Event:** eventDate: 2022-09-06; **Record Level:** collectionID: DISAFAc
- **Type status:** Other material. **Occurrence:** recordedBy: Ivan Albertin; individualCount: 2; sex: females; lifeStage: adult; occurrenceID: 8F184F45-38F4-5205-A199-042A293A8935; **Taxon:** scientificName: Osbornellusauronitens (Provancher, 1889); order: Hemiptera; family: Cicadellidae; genus: Osbornellus; specificEpithet: auronitens; scientificNameAuthorship: (Provancher, 1889); **Location:** country: Italy; countryCode: IT; stateProvince: Piedmont; county: Biella; municipality: Cavagliŕ; decimalLatitude: 45.418575; decimalLongitude: 8.124102; geodeticDatum: WGS84; **Identification:** identifiedBy: Francesca Cinquatti; **Event:** eventDate: 2022-09-06; **Record Level:** collectionID: DISAFAc
- **Type status:** Other material. **Occurrence:** recordedBy: Ivan Albertin; individualCount: 5; sex: females; lifeStage: adult; occurrenceID: DDF997CB-6787-5750-80A7-43EB3364CC9F; **Taxon:** scientificName: Osbornellusauronitens (Provancher, 1889); order: Hemiptera; family: Cicadellidae; genus: Osbornellus; specificEpithet: auronitens; scientificNameAuthorship: (Provancher, 1889); **Location:** country: Italy; countryCode: IT; stateProvince: Piedmont; county: Torino; municipality: Borgomasino; decimalLatitude: 45.365531; decimalLongitude: 7.989988; geodeticDatum: WGS84; **Identification:** identifiedBy: Francesca Cinquatti; **Event:** eventDate: 2022-09-13; **Record Level:** collectionID: DISAFAc
- **Type status:** Other material. **Occurrence:** recordedBy: Ivan Albertin; individualCount: 1; sex: female; lifeStage: adult; occurrenceID: 9280CF28-C412-5ECB-A7C9-7396C0C1DCC0; **Taxon:** scientificName: Osbornellusauronitens (Provancher, 1889); order: Hemiptera; family: Cicadellidae; genus: Osbornellus; specificEpithet: auronitens; scientificNameAuthorship: (Provancher, 1889); **Location:** country: Italy; countryCode: IT; stateProvince: Piedmont; county: Torino; municipality: Albiano d'Ivrea; decimalLatitude: 45.433117; decimalLongitude: 7.965388; geodeticDatum: WGS84; **Identification:** identifiedBy: Francesca Cinquatti; **Event:** eventDate: 2022-09-13; **Record Level:** collectionID: DISAFAc
- **Type status:** Other material. **Occurrence:** recordedBy: Federico Lessio; individualCount: 1; sex: female; lifeStage: adult; occurrenceID: 25235923-BD95-54F7-A2FA-62937BD233A3; **Taxon:** scientificName: Osbornellusauronitens (Provancher, 1889); order: Hemiptera; family: Cicadellidae; genus: Osbornellus; specificEpithet: auronitens; scientificNameAuthorship: (Provancher, 1889); **Location:** country: Italy; countryCode: IT; stateProvince: Piedmont; county: Biella; municipality: Cavagliŕ; decimalLatitude: 45.418575; decimalLongitude: 8.124102; geodeticDatum: WGS84; **Identification:** identifiedBy: Francesca Cinquatti; **Event:** eventDate: 2022-09-14; **Record Level:** collectionID: DISAFAc
- **Type status:** Other material. **Occurrence:** recordedBy: Federico Lessio; individualCount: 1; sex: female; lifeStage: adult; occurrenceID: C04D64F5-6162-5C74-A171-E28012531DFF; **Taxon:** scientificName: Osbornellusauronitens (Provancher, 1889); order: Hemiptera; family: Cicadellidae; genus: Osbornellus; specificEpithet: auronitens; scientificNameAuthorship: (Provancher, 1889); **Location:** country: Italy; countryCode: IT; stateProvince: Piedmont; county: Torino; municipality: Palazzo Canavese; decimalLatitude: 45.461246; decimalLongitude: 7.990519; geodeticDatum: WGS84; **Identification:** identifiedBy: Francesca Cinquatti; **Event:** eventDate: 2022-09-14; **Record Level:** collectionID: DISAFAc
- **Type status:** Other material. **Occurrence:** recordedBy: Federico Lessio; individualCount: 2; sex: 1 male, 1 female; lifeStage: adult; occurrenceID: E6B9EA39-B840-513F-B9A4-FF91254332F8; **Taxon:** scientificName: Osbornellusauronitens (Provancher, 1889); order: Hemiptera; family: Cicadellidae; genus: Osbornellus; specificEpithet: auronitens; scientificNameAuthorship: (Provancher, 1889); **Location:** country: Italy; countryCode: IT; stateProvince: Piedmont; county: Torino; municipality: Borgomasino; decimalLatitude: 45.365531; decimalLongitude: 7.989988; geodeticDatum: WGS84; **Identification:** identifiedBy: Francesca Cinquatti; **Event:** eventDate: 2022-09-13; **Record Level:** collectionID: DISAFAc
- **Type status:** Other material. **Occurrence:** recordedBy: Federico Lessio; individualCount: 1; sex: female; lifeStage: adult; occurrenceID: DCAC8472-2EC5-5467-8889-9D4B1DDB0B52; **Taxon:** scientificName: Osbornellusauronitens (Provancher, 1889); order: Hemiptera; family: Cicadellidae; genus: Osbornellus; specificEpithet: auronitens; scientificNameAuthorship: (Provancher, 1889); **Location:** country: Italy; countryCode: IT; stateProvince: Piedmont; county: Torino; municipality: Caluso; decimalLatitude: 45.307469; decimalLongitude: 7.910311; geodeticDatum: WGS84; **Identification:** identifiedBy: Francesca Cinquatti; **Event:** eventDate: 2022-08-31; **Record Level:** collectionID: DISAFAc
- **Type status:** Other material. **Occurrence:** recordedBy: Federico Lessio; individualCount: 1; sex: female; lifeStage: adult; occurrenceID: B2B5F65E-357A-574B-8D3D-C115F96F13B6; **Taxon:** scientificName: Osbornellusauronitens (Provancher, 1889); order: Hemiptera; family: Cicadellidae; genus: Osbornellus; specificEpithet: auronitens; scientificNameAuthorship: (Provancher, 1889); **Location:** country: Italy; countryCode: IT; stateProvince: Piedmont; county: Torino; municipality: Cossano Canavese; decimalLatitude: 45.382650; decimalLongitude: 7.996746; geodeticDatum: WGS84; **Identification:** identifiedBy: Francesca Cinquatti; **Event:** eventDate: 2022-09-20; **Record Level:** collectionID: DISAFAc
- **Type status:** Other material. **Occurrence:** recordedBy: Federico Lessio; individualCount: 1; sex: female; lifeStage: adult; occurrenceID: 7931562B-4E27-537D-B675-E9C140497E13; **Taxon:** scientificName: Osbornellusauronitens (Provancher, 1889); order: Hemiptera; family: Cicadellidae; genus: Osbornellus; specificEpithet: auronitens; scientificNameAuthorship: (Provancher, 1889); **Location:** country: Italy; countryCode: IT; stateProvince: Piedmont; county: Torino; municipality: Cossano Canavese; decimalLatitude: 45.382650; decimalLongitude: 7.996746; geodeticDatum: WGS84; **Identification:** identifiedBy: Francesca Cinquatti; **Event:** eventDate: 2022-09-08; **Record Level:** collectionID: DISAFAc
- **Type status:** Other material. **Occurrence:** recordedBy: Federico Lessio; individualCount: 1; sex: female; lifeStage: adult; occurrenceID: CA4437CB-3259-5B55-B0EA-B4D9E672C1B1; **Taxon:** scientificName: Osbornellusauronitens (Provancher, 1889); order: Hemiptera; family: Cicadellidae; genus: Osbornellus; specificEpithet: auronitens; scientificNameAuthorship: (Provancher, 1889); **Location:** country: Italy; countryCode: IT; stateProvince: Piedmont; county: Biella; municipality: Cerrione; decimalLatitude: 45.466442; decimalLongitude: 8.070268; geodeticDatum: WGS84; **Identification:** identifiedBy: Francesca Cinquatti; **Event:** eventDate: 2022-09-20; **Record Level:** collectionID: DISAFAc
- **Type status:** Other material. **Occurrence:** recordedBy: Federico Lessio; individualCount: 1; sex: female; lifeStage: adult; occurrenceID: 33CFB038-0959-5684-B6C1-10C16CF8056F; **Taxon:** scientificName: Osbornellusauronitens (Provancher, 1889); order: Hemiptera; family: Cicadellidae; genus: Osbornellus; specificEpithet: auronitens; scientificNameAuthorship: (Provancher, 1889); **Location:** country: Italy; countryCode: IT; stateProvince: Piedmont; county: Biella; municipality: Cerrione; decimalLatitude: 45.466442; decimalLongitude: 8.070268; geodeticDatum: WGS84; **Identification:** identifiedBy: Francesca Cinquatti; **Event:** eventDate: 2022-09-20; **Record Level:** collectionID: DISAFAc
- **Type status:** Other material. **Occurrence:** recordedBy: Federico Lessio; individualCount: 1; sex: female; lifeStage: adult; occurrenceID: 0814D56C-8E5E-5647-9AB2-9B023CE192C9; **Taxon:** scientificName: Osbornellusauronitens (Provancher, 1889); order: Hemiptera; family: Cicadellidae; genus: Osbornellus; specificEpithet: auronitens; scientificNameAuthorship: (Provancher, 1889); **Location:** country: Italy; countryCode: IT; stateProvince: Piedmont; county: Torino; municipality: Maglione; decimalLatitude: 45.365329; decimalLongitude: 8.005953; geodeticDatum: WGS84; **Identification:** identifiedBy: Francesca Cinquatti; **Event:** eventDate: 2022-09-15; **Record Level:** collectionID: DISAFAc
- **Type status:** Other material. **Occurrence:** recordedBy: Federico Lessio; individualCount: 1; sex: female; lifeStage: adult; occurrenceID: FE299B1B-E6FB-5238-BA46-D9E3D532B07D; **Taxon:** scientificName: Osbornellusauronitens (Provancher, 1889); order: Hemiptera; family: Cicadellidae; genus: Osbornellus; specificEpithet: auronitens; scientificNameAuthorship: (Provancher, 1889); **Location:** country: Italy; countryCode: IT; stateProvince: Piedmont; county: Torino; municipality: Piverone; decimalLatitude: 45.438961; decimalLongitude: 8.027062; geodeticDatum: WGS84; **Identification:** identifiedBy: Francesca Cinquatti; **Event:** eventDate: 2022-09-14; **Record Level:** collectionID: DISAFAc
- **Type status:** Other material. **Occurrence:** recordedBy: Federico Lessio; individualCount: 1; sex: female; lifeStage: adult; occurrenceID: 392A1C2F-C5D8-5281-9B96-17635455DFC9; **Taxon:** scientificName: Osbornellusauronitens (Provancher, 1889); order: Hemiptera; family: Cicadellidae; genus: Osbornellus; specificEpithet: auronitens; scientificNameAuthorship: (Provancher, 1889); **Location:** country: Italy; countryCode: IT; stateProvince: Piedmont; county: Torino; municipality: Caluso; decimalLatitude: 45.307469; decimalLongitude: 7.910311; geodeticDatum: WGS84; **Identification:** identifiedBy: Francesca Cinquatti; **Event:** eventDate: 2022-09-15; **Record Level:** collectionID: DISAFAc
- **Type status:** Other material. **Occurrence:** recordedBy: Federico Lessio; individualCount: 1; sex: female; lifeStage: adult; occurrenceID: 4893E74F-00A7-5034-A0A7-935B78CBC12D; **Taxon:** scientificName: Osbornellusauronitens (Provancher, 1889); order: Hemiptera; family: Cicadellidae; genus: Osbornellus; specificEpithet: auronitens; scientificNameAuthorship: (Provancher, 1889); **Location:** country: Italy; countryCode: IT; stateProvince: Piedmont; county: Torino; municipality: Maglione; decimalLatitude: 45.365329; decimalLongitude: 8.005953; geodeticDatum: WGS84; **Identification:** identifiedBy: Francesca Cinquatti; **Event:** eventDate: 2022-09-15; **Record Level:** collectionID: DISAFAc
- **Type status:** Other material. **Occurrence:** recordedBy: Federico Lessio; individualCount: 2; sex: 1 male, 1 female; lifeStage: adult; occurrenceID: 645724D7-7CA8-50C9-BD7B-B0502B49B0CF; **Taxon:** scientificName: Osbornellusauronitens (Provancher, 1889); order: Hemiptera; family: Cicadellidae; genus: Osbornellus; specificEpithet: auronitens; scientificNameAuthorship: (Provancher, 1889); **Location:** country: Italy; countryCode: IT; stateProvince: Piedmont; county: Torino; municipality: Maglione; decimalLatitude: 45.365329; decimalLongitude: 8.005953; geodeticDatum: WGS84; **Identification:** identifiedBy: Francesca Cinquatti; **Event:** eventDate: 2022-09-15; **Record Level:** collectionID: DISAFAc
- **Type status:** Other material. **Occurrence:** recordedBy: Federico Lessio; individualCount: 7; sex: females; lifeStage: adult; occurrenceID: A485D1BA-94D8-538D-BF65-16253A936EEE; **Taxon:** scientificName: Osbornellusauronitens (Provancher, 1889); order: Hemiptera; family: Cicadellidae; genus: Osbornellus; specificEpithet: auronitens; scientificNameAuthorship: (Provancher, 1889); **Location:** country: Italy; countryCode: IT; stateProvince: Piedmont; county: Torino; municipality: Borgomasino/Maglione; decimalLatitude: 45.356061; decimalLongitude: 8.000192; geodeticDatum: WGS84; **Identification:** identifiedBy: Francesca Cinquatti; **Event:** eventDate: 2022-09-15; **Record Level:** collectionID: DISAFAc
- **Type status:** Other material. **Occurrence:** recordedBy: Federico Lessio; individualCount: 3; sex: males; lifeStage: adult; occurrenceID: 84523460-62EA-5117-8A0D-E0061648F10D; **Taxon:** scientificName: Osbornellusauronitens (Provancher, 1889); order: Hemiptera; family: Cicadellidae; genus: Osbornellus; specificEpithet: auronitens; scientificNameAuthorship: (Provancher, 1889); **Location:** country: Italy; countryCode: IT; stateProvince: Piedmont; county: Torino; municipality: Borgomasino; decimalLatitude: 45.365531; decimalLongitude: 7.989988; geodeticDatum: WGS84; **Identification:** identifiedBy: Francesca Cinquatti; **Event:** eventDate: 2022-09-13; **Record Level:** collectionID: DISAFAc
- **Type status:** Other material. **Occurrence:** recordedBy: Federico Lessio; individualCount: 1; sex: female; lifeStage: adult; occurrenceID: E3D1CBB7-7494-5917-ABCE-CD293BFD5E3B; **Taxon:** scientificName: Osbornellusauronitens (Provancher, 1889); order: Hemiptera; family: Cicadellidae; genus: Osbornellus; specificEpithet: auronitens; scientificNameAuthorship: (Provancher, 1889); **Location:** country: Italy; countryCode: IT; stateProvince: Piedmont; county: Torino; municipality: Viverone; decimalLatitude: 45.428387; decimalLongitude: 8.066156; geodeticDatum: WGS84; **Identification:** identifiedBy: Francesca Cinquatti; **Event:** eventDate: 2022-10-12; **Record Level:** collectionID: DISAFAc
- **Type status:** Other material. **Occurrence:** recordedBy: Federico Lessio; individualCount: 1; sex: female; lifeStage: adult; occurrenceID: D8E006C5-0826-5C9A-B279-CB8A9C5AD111; **Taxon:** scientificName: Osbornellusauronitens (Provancher, 1889); order: Hemiptera; family: Cicadellidae; genus: Osbornellus; specificEpithet: auronitens; scientificNameAuthorship: (Provancher, 1889); **Location:** country: Italy; countryCode: IT; stateProvince: Piedmont; county: Torino; municipality: Borgomasino; decimalLatitude: 45.365531; decimalLongitude: 7.989988; geodeticDatum: WGS84; **Identification:** identifiedBy: Francesca Cinquatti; **Event:** eventDate: 2022-10-12; **Record Level:** collectionID: DISAFAc
- **Type status:** Other material. **Occurrence:** recordedBy: Federico Lessio; individualCount: 1; sex: female; lifeStage: adult; occurrenceID: C8A22D72-AFE2-576C-8103-42FD8D928325; **Taxon:** scientificName: Osbornellusauronitens (Provancher, 1889); order: Hemiptera; family: Cicadellidae; genus: Osbornellus; specificEpithet: auronitens; scientificNameAuthorship: (Provancher, 1889); **Location:** country: Italy; countryCode: IT; stateProvince: Piedmont; county: Torino; municipality: Borgomasino; decimalLatitude: 45.365531; decimalLongitude: 7.989988; geodeticDatum: WGS84; **Identification:** identifiedBy: Francesca Cinquatti; **Event:** eventDate: 2022-10-12; **Record Level:** collectionID: DISAFAc
- **Type status:** Other material. **Occurrence:** recordedBy: Federico Lessio; individualCount: 1; sex: female; lifeStage: adult; occurrenceID: EF713DFE-2653-5321-8763-F16291E47C0E; **Taxon:** scientificName: Osbornellusauronitens (Provancher, 1889); order: Hemiptera; family: Cicadellidae; genus: Osbornellus; specificEpithet: auronitens; scientificNameAuthorship: (Provancher, 1889); **Location:** country: Italy; countryCode: IT; stateProvince: Piedmont; county: Torino; municipality: Viverone; decimalLatitude: 45.428387; decimalLongitude: 8.066156; geodeticDatum: WGS84; **Identification:** identifiedBy: Francesca Cinquatti; **Event:** eventDate: 2022-10-12; **Record Level:** collectionID: DISAFAc

#### Diagnosis

**Figure 1.**
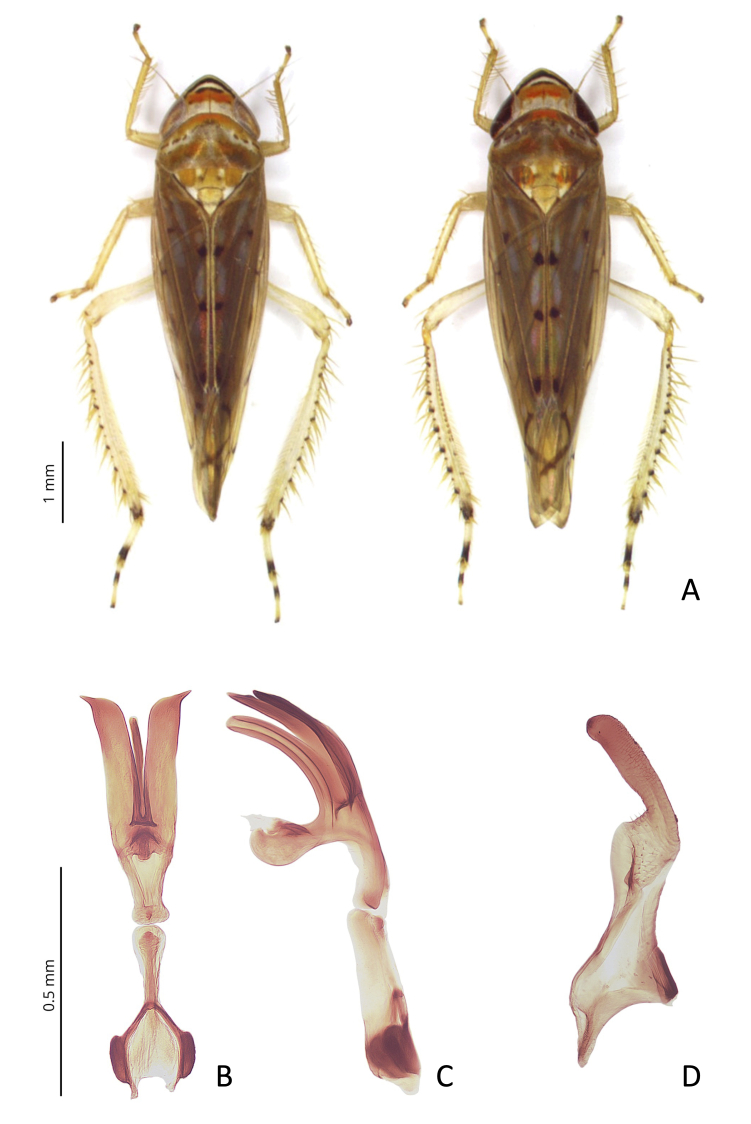
*Osbornellusauronitens* (Provancher, 1889): **A** dorsal habitus, male (left) and female (right). Specimens collected in Lombardy in 2015; **B** aedeagus and connective, ventral view; **C** aedeagus and connective, lateral view; **D** left stylus, ventral view.

Body length 5.5-6 mm; base colour of the integuments pale yellow. Vertex triangular, with an orange median transverse band and an incomplete brownish-black pre-apical line; vertex-face transition with a supra-ocellar transverse black line limiting anterior margin and a second submarginal black line; face pale, with or without brownish transverse lines. Pronotum with two orange transverse bands. Forewings elongate, tawny, with lighter areoles; inner margin of clavus with three dark spots; median, radial and subapical cells sometimes with pale brownish markings; veins brownish, whitish on clavus; radial vein with three branches (Fig. 1). Male valve rounded; subgenital plates elongate, with slender filamentous distal portion common to most species of the genus; style apical process wide, with sides almost parallel, apex rounded slightly concave on outer margin. Aedeagus curved dorsally, with a pair of broad processes arising ventrolaterally just before base of shaft, flaring slightly, extending just beyond apex of shaft in dorsally pointed apices (Fig. 1). A more detailed description is provided by Beamer (1937) and Trivellone et al. (2017).

#### Remarks

Amongst the leafhoppers so far reported for Europe, *O.auronitens* is morphologically similar to the invasive and widely distributed *Scaphoideustitanus* Ball (Trivellone et al. 2017); from the latter, *O.auronitens* can be easily distinguished by the presence of the incomplete brownish-black pre-apical line on vertex (absent in *S.titanus*), a lighter colouration of forewings with fewer brown markings on cells, three distinct dark spots on inner margin of clavus and the radial vein with three branches instead of five as on *S.titanus*, as well as by the male genital characters.

## Analysis

### DNA barcoding and phylogenetic analysis

**Figure 2.**
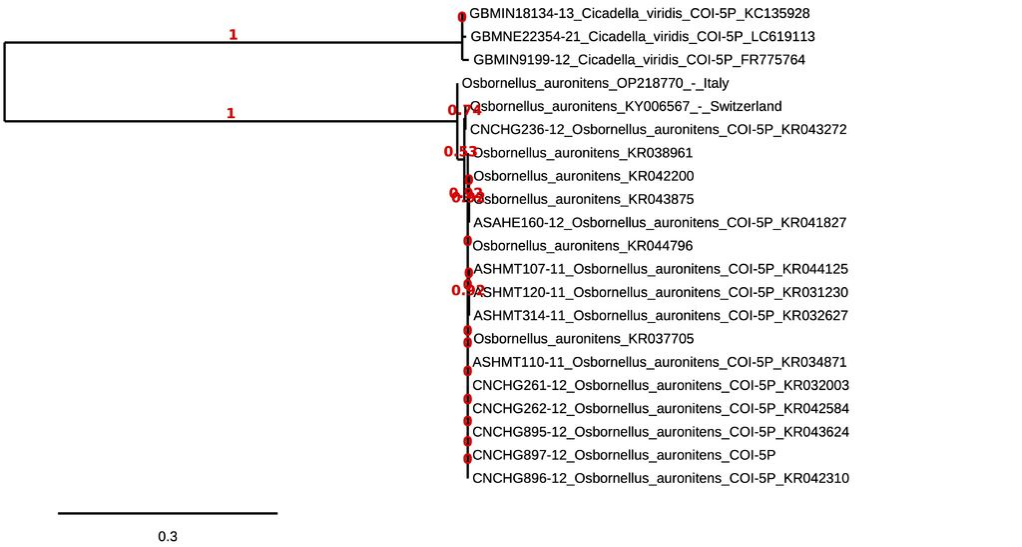
Maximum Likelihood phylogenetic reconstruction of *Osbornellusauronitens* COX1 sequences.

The DNA barcoding resulted in a sequence of 648 bp deposited in GenBank as OP218770. The phylogenetic reconstruction, including *Cicadellaviridis* as the outgroup, resulted in the consensus tree shown in Fig. 2. The topology of the whole tree was highly supported with the node separating the Italian sequence from the Swiss and the American sequences showing an output of 100% (given as 1) for the Approximate likelihood ratio test. The Italian barcode of *O.auronitens* showed a genetic distance of 0.011 from the Swiss sequence and a 0.015 mean distance from the North American pool; in addition, the barcode from Switzerland showed a mean genetic distance from the North American sequences of 0.008.

### Phenology

Our preliminary observation, based on specimens recollected in vineyards and orchards, also showed how *O.auronitens* adults may occur during late summer and early autumn (July-October), with males appearing and reaching their peak of activity almost one month earlier than females and with a *sex ratio* (Males/Females) of 0.64 to 1. However, these findings should be considered with caution.

## Discussion

*Osbornellusauronitens* (Provancher, 1889), the type species of the genus, is a Cicadellidae Deltocephalinae distributed in the north-eastern part of the North American continent throughout Canada and the United States (Beamer 1937). Specifically regarding Europe, this species has been recently recorded for Switzerland (Canton Ticino) in 2016 and 2017 (Trivellone et al. 2017, Trivellone et al. 2022); therefore, the data presented here constitute the first record of this non-native species for Italy. The discovery of *O.auronitens* in a particularly large area and during different years would suggest that Italian populations might not be the result of a recent introduction (as suggested by the 2015 record), but simply either remained undetected or misidentified until now. It remains unclear if the two populations from Piedmont and Lombardy are related to each other. In addition, although the short geographical distance between the Swiss and Italian localities would suggest that the Italian population could be the result of a range expansion of the other one (or vice versa), genetic data would seem to suggest that the two populations might be the result of two independent introductions. Species records from Piedmont, in addition to those from Switzerland, suggest how *O.auronitens* could be capable of colonising vineyards and fruit orchards. However, although *O.auronitens* is considered a potential vector of phytoplasmas in the United States (Beanland et al. 2006) and it was initially suggested as plausible vector also in Europe (Trivellone et al. 2017), to date, there is no evidence of any phytosanitary relevance of this species (Trivellone et al. 2022). At present, *O.auronitens* is not a regulated pest in the EU; therefore, it does not undergo either specific surveillance or quarantine measures and its interception is not a particular concern. However, further research is needed about the relationships between *O.auronitens*, host plants and plant pathogens, such as phytoplasmas.

## Supplementary Material

XML Treatment for Osbornellus (Osbornellus) auronitens
